# Evaluation of the Antimicrobial Potential of *Toxocara canis* Adult and Larval Somatic and ES Antigens Against *Staphylococcus aureus*, *Enterococcus faecalis*, *Escherichia coli*, and *Salmonella enterica*

**DOI:** 10.1155/jotm/6677365

**Published:** 2025-07-24

**Authors:** Chia-Kwung Fan, Yi-Hsuan Ma, Hon-Ian Lei, Yu-Chu Chang, Yu-Heng Chou, Chia-Mei Chou

**Affiliations:** ^1^Department of Molecular Parasitology and Tropical Diseases, School of Medicine, College of Medicine, Taipei Medical University, 250 Wu-Xing Street, Taipei 11031, Taiwan; ^2^Center for International Tropical Medicine Research, College of Medicine, Taipei Medical University, 250 Wu-Xing Street, Taipei 11031, Taiwan; ^3^Department of Biochemistry and Molecular Cell Biology, School of Medicine, College of Medicine, Taipei Medical University, 250 Wu-Xing Street, Taipei 11031, Taiwan; ^4^Department of Protein Evolution, Max Planck Institute for Biology Tübingen, Tübingen 72076, Germany

**Keywords:** antimicrobial activity, bacterial pathogens, excretory-secretory antigens, somatic antigens, *Toxocara canis*

## Abstract

**Background:** Antimicrobial resistance (AMR) is a major global threat to public health and development. The emergence of drug-resistant pathogens compromises the effectiveness of current treatments and necessitates the discovery of new antimicrobial agents.

**Objective:** This study aims to evaluate the antimicrobial potential of *Toxocara canis* adult and larval somatic and excretory-secretory (ES) antigens against common bacterial pathogens, including *Staphylococcus aureus*, *Enterococcus faecalis*, *Escherichia coli*, and *Salmonella enterica*.

**Methods:** Adult *Toxocara canis* worms were collected and dissected to obtain somatic antigenic proteins. Larval somatic and ES antigenic proteins were prepared from hatched eggs. The antimicrobial activity of these antigens was evaluated using susceptibility assays and minimum inhibitory concentration (MIC) and minimum bactericidal concentration (MBC) tests. High-performance liquid chromatography (HPLC) was used to investigate the possible bactericidal properties in comparison with standard tetracycline antibiotics.

**Results:** The somatic antigenic proteins of adult and larval *T. canis* worms and larval ES antigens showed effective antimicrobial potential against *Staphylococcus aureus*, but not against the other 3 bacteria. HPLC analysis suggested that the bactericidal properties of these proteins may be close to that of tetracycline antibiotics because of the similar retention time.

**Conclusion:** The preliminary study provides evidence of the antimicrobial properties of different stages of *T. canis* antigens, offering potential new solutions to combat AMR. Further research is needed to elucidate the mechanisms of action and assess the clinical applications of these antigenic proteins.

## 1. Background

Antimicrobial resistance (AMR) is a growing global concern, endangering public health and hindering development efforts worldwide. In 2019, bacterial AMR was responsible for approximately 1.27 million deaths and played a role in 4.95 million deaths globally. Antimicrobial agents are essential to modern health care, yet the increasing prevalence of drug-resistant microbes threatens our capacity to manage routine infections and conduct vital medical interventions, such as cancer treatments, cesarean deliveries, hip surgeries, organ transplants, and other critical procedures [1]. The rise of drug-resistant infections also affects animal and plant health, reducing agricultural yields and posing risks to food security. In response, the World Health Organization (WHO), the World Organization for Animal Health (OIE), and the Food and Agriculture Organization (FAO) have joined forces to tackle this issue [2].

Bacteria have different levels of sensitivity to various medications and constantly mutate to ensure their survival. Improper use of medications not only fails to eliminate the infection but also encourages microorganisms to develop resistance to drugs [3–5]. Presently, the group of bacteria referred to as ESKAPE pathogens—which includes *Enterococcus faecium*, *Staphylococcus aureus*, *Klebsiella pneumoniae*, *Acinetobacter baumannii*, *Pseudomonas aeruginosa*, and *Enterobacter* species—are the primary culprits behind hospital-acquired infections on a global scale. Recent data indicate that several of these pathogens have gained resistance to multiple antibiotics. For instance, *S. aureus* has evolved into a multidrug-resistant organism, linked to serious health issues such as implant-associated infections, endocarditis, pneumonia, mastitis, osteomyelitis, and sepsis [6]. *Enterococci*, another major cause of hospital-acquired infections, contribute to considerable illness and death. *Enterococcus faecalis*, the more prevalent and virulent species, is known for causing severe high-inoculum infections like infective endocarditis, often associated with cardiac surgery, with mortality rates that have remained constant over the past 3 decades [7].

While most *Escherichia coli* strains are harmless and commonly found in the human gut, some virulent strains can lead to a range of diseases, such as gastroenteritis, urinary tract infections, neonatal meningitis, hemorrhagic colitis, and Crohn's disease. In rare instances, these harmful strains can also cause intestinal tissue death and perforation, potentially resulting in conditions like hemolytic uremic syndrome, peritonitis, mastitis, sepsis, and gram-negative pneumonia [8].

*Salmonella enterica* is a gram-negative, rod-shaped, flagellated bacterium that belongs to the *Salmonella* genus and is facultatively anaerobic. Food contaminated with *S. enterica*, often from cattle and poultry, is the leading cause of salmonellosis. However, other animals, including domestic cats, can also transmit this infection to humans. This bacterium primarily inhabits the human intestinal tract and can also be present in food, soil, bedding, litter, and feces. Poultry serves as the main reservoir for *S. enterica*, with around 70% of human cases linked to the consumption of contaminated eggs, chicken, or turkey. The pathogen's secreted proteins play a vital role in its ability to cause infectious diseases [9].

Antimicrobial peptides (AMPs), also known as host defense peptides (HDPs), are crucial components of the innate immune system across various life forms. These peptides exploit the fundamental differences between prokaryotic and eukaryotic cells, making them effective broad-spectrum antimicrobials with potential therapeutic applications. Certain AMPs have demonstrated the ability to kill both gram-negative and gram-positive bacteria [10], as well as enveloped viruses, fungi, and even cancerous or transformed cells [11]. Research indicates that parasitic nematodes in the mammalian small intestine can survive in hydrolytic environments, resist host immune responses, and thrive in microbial-rich surroundings. The ability of intestinal nematodes to defend themselves against gut microbiota appears crucial for their survival [12]. For example, it has been observed that the intestinal cells and body cavity fluid of adult *Ascaris suum* can secrete an antibacterial factor (ASABF) that can inhibit *Escherichia coli* or kill *S. aureus* [13]. Further studies revealed that the molecular structure and bactericidal mechanism of ASABF are like those of defensin, which is secreted by insects [14]. Additionally, recombinant ASABF peptides have been shown to effectively kill both gram-positive and gram-negative bacteria in vitro [15]. *Toxocara canis* is a parasitic nematode found in the intestines of canids. *T. canis* and *A. suum* are parasitic nematodes that inhabit similar intestinal environments in their respective definitive hosts and belong to the same family, Ascarididae, although they are classified under different genera [16]. However, it remains uncertain whether *T. canis*—either in its adult or larval stages—or the excretory-secretory product (ESP) produced by the larvae, possess bacteriostatic or bactericidal properties, as no studies have explored this. This study aimed to assess the inhibitory and bactericidal effects on gram-positive bacteria, specifically *S. aureus* and *Enterococcus faecalis*, as well as gram-negative bacteria, including *E. coli* and *S. enterica*, by examining various organs of the adult *T. canis* worm, the larvae, and their excretory-secretory (ES) proteins.

## 2. Methods

### 2.1. Collection and Dissection of Adult *Toxocara canis* Worms

Adult *Toxocara canis* worms were obtained from the feces of puppies at the Neihu Stray Animal Protection Shelter in Taipei following the administration of a single mebendazole tablet. If adult worms were detected, they were thoroughly cleaned by repeated washing with phosphate-buffered saline (PBS) (0.2 M, pH 7.4) and confirmed under a dissecting microscope. Each worm was then dissected along the body wall through the anus, and the fluid, intestines, genitalia, and body wall were carefully separated based on sex. The collected samples were stored at −80°C until they were ready for protein extraction.

### 2.2. Egg Culture

Infective embryonated eggs were cultured following a modified version of the method described by Fan et al. [17]. Female worms were dissected, and the uterus was placed in 10 mL of a 1% sodium hypochlorite solution, stirred, and incubated for 5 min at room temperature. The volume was then increased to 20 mL with distilled water. The mixture was passed through gauze to remove any debris and centrifuged at 1500 rpm for 5 min. After discarding the supernatant, the pellet was washed twice with water and once with formalin. The eggs were transferred to a 250-mL Erlenmeyer flask containing formalin, ensuring the liquid level was about 1 cm deep. The flask was sealed with paraffin and kept at room temperature for 8–9 weeks, with gentle agitation once a week. The eggs were stored at 4°C for 14–15 months and washed with water before use according to our previous study [17].

### 2.3. Preparation of *T. canis* Larval ES Antigens

An identical protocol was employed in our earlier research [18]. When larvae were needed, embryonated eggs were hatched under sterile conditions, rinsed with sterile PBS, resuspended in sterile 1% (w/v) sodium hypochlorite, and incubated for 30 min at 37°C in an atmosphere containing 5% CO_2_. Following several washes in sterile PBS with triple antibiotics (100 IU of penicillin, 10 μg streptomycin, and 2 μg/mL amphotericin B; MP Biomedicals, CA, USA), the larvae were resuspended in RPMI-1640 medium (cat. no. 11875093, Thermo Fisher Scientific, MA, USA) containing the same concentrations of the triple antibiotics. Motile larvae were collected using a modified Baermann apparatus placed in an atmosphere containing 5% CO_2_ for 12 h at 37°C.

The larvae were then transferred to 50-mL tissue culture flasks containing 10 mL fresh RPMI-1640 medium and antibiotics, yielding 10^4^ larvae/mL, and incubated in an atmosphere containing 5% CO_2_ at 37°C. The supernatant medium from each culture containing ES antigens (TES) was collected weekly for 3–4 weeks (and replaced with fresh medium). These samples were pooled and centrifuged to remove debris. The resulting supernatant was sterilized by filtration through a 0.2-μm pore membrane and then dialyzed (with a molecular weight cutoff of 6000–8000 kDa) against PBS at 4°C for 12 h or until no phenol red was observed in the medium. Protein concentration was determined by the Bradford method, followed by lyophilization (Labconco, Kansas City, MO, USA). TES samples were stored at −80°C until use.

### 2.4. Preparation of Somatic Antigens From *T. canis* Larvae and Adults

Lyophilized *T. canis* larvae, along with the intestines, genitalia, and body wall from the adults, were homogenized using a Teflon homogenizer. Soluble antigens were extracted in PBS at 4°C for 24 h, following our previous protocol [19]. The body fluid (BF) and homogenate of somatic antigens from larvae, body wall, intestines, and male or female genitals were then centrifuged at 10,000 rpm for 20 min at 4°C. Supernatant samples obtained from the fluid, cuticle, and intestine of adult *T. canis* were designated as TAF, TAC, and TAI, respectively. Supernatants from the male and female genital organs were labeled TMG and TFG, while the somatic antigens derived from *T. canis* larvae were designated TLS. Protein content of the dialyzate was estimated using the Bradford method, then concentrated by lyophilization (FOM-2, UNISS, BioLion Technology Co., Ltd., Taichung, Taiwan), and stored at −80°C until use.

### 2.5. Bacterial Strain Maintenance

*Staphylococcus aureus* (ATCC 6538P), *Enterococcus faecalis* (ATCC 14506), *Escherichia coli* (JM101; ATCC 33876), and *Salmonella enterica* subsp. (ATCC 14028) were from ATCC, USA. Difco™ Lactobacilli MRS Broth (no.288130, Creative Life Science Co., Ltd, New Taipei City, Taiwan) for minimal inhibitory concentration (MIC) and Difco™ Mueller Hinton (MH) agar for disk diffusion technique and minimal bactericidal concentration (MBC) (no. 225250) were purchased from Creative Life Science Company, New Taipei City, Taiwan.

### 2.6. Antibacterial Activity and Susceptibility Assays

A simple and effective way to evaluate how well an antibiotic works against specific pathogenic bacteria in a controlled laboratory setting is by testing the antibiotic susceptibility of bacterial strains [4, 5]. The microdilution method was employed to assess the antimicrobial spectrum against the studied bacterial species following CLSI guidelines [20]. In brief, overnight bacterial suspensions matching the 0.5 McFarland standard were diluted to 1.5 × 10^5^ CFU·mL^−1^ to test the antimicrobial effects of TAI, TAF, TAC, TMG, TFG, TLS, and TES (concentration range: 3397–13,390 μg·mL^−1^) in nutrient broth medium in 96 ultra-clear round-bottom microplates (Cat. No. BL6032, Basic Life Science Inc. Taiwan). The plates were incubated at 37°C for 24 h while shaking at 200 rpm. For the susceptibility test, the bacterial inoculum was adjusted to the 0.5 McFarland standard and 100 μL was inoculated onto MH agar. Then, 20 μL of TAI, TAF, TAC, TMG, TFG, TLS, or TES (a twofold increase of the MIC for each bacterial species) was added to the disks (Φ = 8 mm). Plates were incubated at 37°C for 24 h, and the zone of inhibition was measured. Additionally, tetracycline (Te-30), gentamycin (Ge-10), and chloramphenicol (C-30) were used as controls. All experiments were repeated three times on different days. The lowest concentration at which no visible bacterial growth was detected was defined as the minimum inhibitory concentration (MIC). The highest concentration at which no colony growth was observed on the agar plate after incubation at 37°C for another 24 h was considered the minimal bactericidal concentration (MBC).

### 2.7. High-Performance Liquid Chromatography (HPLC)

TAI, TAF, TAC, TMG, TFG, TLS, or TES was fractionated for preliminary isolation of antibacterial activity by using a model L-7000 HPLC system (L-7000 DAD HPLC System, Hitachi High Technologies Corporation, Tokyo, Japan) at the Taipei Medical University according to a method by Abner et al. [21] with a slight modification. Antigenic proteins (150 μL) with antimicrobial potential were mixed with an equal volume of 0.1% trifluoroacetic acid (TFA) and centrifuged at 14,000 rpm for 15 min at room temperature. Then, 200 μL of the clarified supernatant with antigenic proteins with antimicrobial potential was injected onto a C18 reverse phase column (0.8 mm diameter, 250 mm long) packed with Vydak 300A˚ resin (LC Packings, San Francisco, CA). Compounds were eluted by a continuous linear gradient from 5% acetonitrile (ACN) in 0.1% TFA to 99% ACN over 3 h at a flow rate of 0.5 mL/min. The absorbance of the eluate was monitored at 214 nm, and fractions were collected manually as peaks were detected. The eluted fractions were dried in a Savant Speedvac concentrator, reconstituted in 50 μL sterile ultrapure water and filter-sterilized (Spin X columns; Costar) before bioassay for antibacterial activity using the broth microdilution assay. The peak signals of the antimicrobial antigenic proteins were used to compare the retention time with the antibiotic standard (tetracycline hydrochloride, T3383, Sigma-Aldrich, Missouri, USA) to confirm whether the signal of the antigen was similar to the antibiotic standard to explore the possible bactericidal properties.

### 2.8. Preparation of Antimicrobial Proteins From Adult and Larvae by HPLC With a Molecular Weight of Less Than 3 kDa

Antimicrobial proteins eluted from adults and larvae by HPLC were concentrated using a 3 kDa concentrator kit (No. 112550588, NanoSep 3 K Omega, Life Science, Taiwan), and the resulting solutions were used for MIC and MBC testing of *S. aureus*.

## 3. Result

### 3.1. Referent Antibiotic Selection by Susceptibility Testing of *S. aureus*, *E. faecalis*, *S. enterica* subsp., and *E. coli*

**Figure 1:**
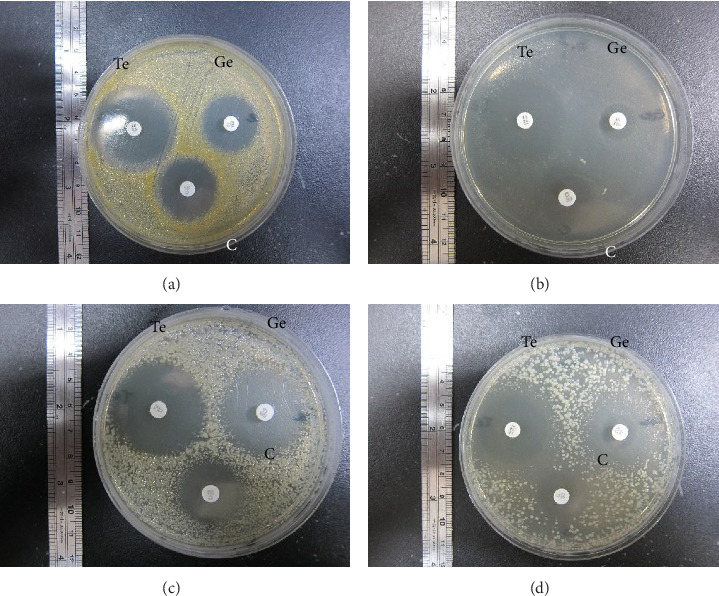
Reference antibiotic selection by susceptibility testing among tetracycline, gentamycin, and chloramphenicol on (a) *Staphylococcus aureus*, (b) *Enterococcus faecalis*, (c) *Escherichia coli*, and (d) *Salmonella enterica* subsp.

Based on the susceptibility test in which the zone ≥ 19 mm was determined as sensitive (S), < 14 mm as resistant (R) and 15∼18 mm as intermediate (I) [22], the 3 broad-spectrum anti-gram (+)/gram (-) antibiotics, that is, tetracycline, gentamycin, and chloramphenicol all pose effectively antimicrobial capacity against *S. aureus* (35, 24, 26 mm), *E. faecalis* (25, 15, 19 mm), *S. enterica* subsp. (33, 25, 20 mm), and *E. coli* (33, 31, 20 mm) (Figures 1(a), 1(b), 1(c), 1(d)). Since the tetracycline showed the largest inhibition zone among the three antibiotics, the tetracycline was selected as the referent antibiotics for the susceptibility test for various antigenic proteins to reduce false positive results.

### 3.2. Susceptibility Testing for Somatic Antigenic Proteins of Adult *T. canis* Worm on *S. aureus*, *E. faecalis*, *S. enterica* subsp., and *E. coli*

**Figure 2:**
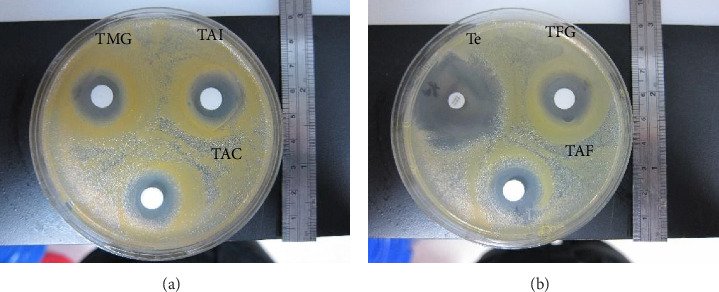
Susceptibility testing for somatic antigenic proteins of adult *Toxocara canis* worm including (a) genitals of male adult *T. canis* (TMG), intestine (TAI), and body wall (TAC) of adult *T. canis* and (b) genitals of female adult *Toxocara canis* (TFG), and body fluid of adult *T. canis* (TAF) on *Staphylococcus aureus*. Te: Tetracycline.

Among the somatic antigenic proteins of adult *T. canis* worm on the antimicrobial potential against the 4 bacteria, all were almost able to show the effective inhibition zone on *S. aureus*, indicating TAC (20 mm, S), TMG (19 mm, S), TFG (20 mm, S), and TAF (20 mm, S) (Figures 2(a), 2(b)); however, the inhibition zone of TAI on *S. aureus* was merely 17 mm (I) (Figure 2(a)). In contrast, they did not show any effective inhibition ability against the other 3 bacteria (data not shown).

### 3.3. Susceptibility Testing for Larval Somatic and ES Antigenic Proteins on *S. aureus*, *E. faecalis*, *S. enterica* subsp., and *E. coli*

**Figure 3:**
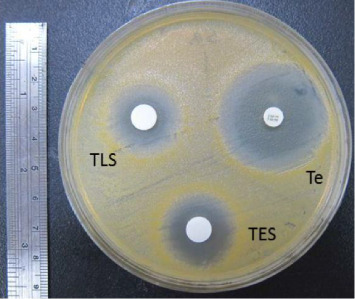
Susceptibility testing for larval somatic (TLS) and excretory-secretary antigenic proteins (TES) of *Toxocara canis* on *Staphylococcus aureus*. Te: Tetracycline.

Similarly, the larval somatic and ES antigenic proteins showed the antimicrobial potential against only on *S. aureus*, indicating TLS (22 mm, S) and TES (21 mm, S) (Figure 3); they did not show any effective inhibition ability against the other 3 bacteria (data not shown).

### 3.4. MIC and MBC Testing of Somatic and ES Antigenic Proteins of Adult and Larval *T. canis* on *S. aureus*

The MIC results of TAI, TAC, TMG, TFG, and TAF on *S. aureus* were 1: 32 (203.13 μg/mL), 1: 64 (101.56 μg/mL), 1: 64 (101.56 μg/mL), 1: 64 (101.56 μg/mL), and 1: 8 (203.13 μg/mL), respectively (Table 1). The MIC results of TLS and TES on *S. aureus* were 1: 64 (41.90 μg/mL) and 1: 16 (167.50 μg/mL), respectively (Table 1).

**Figure 4:**
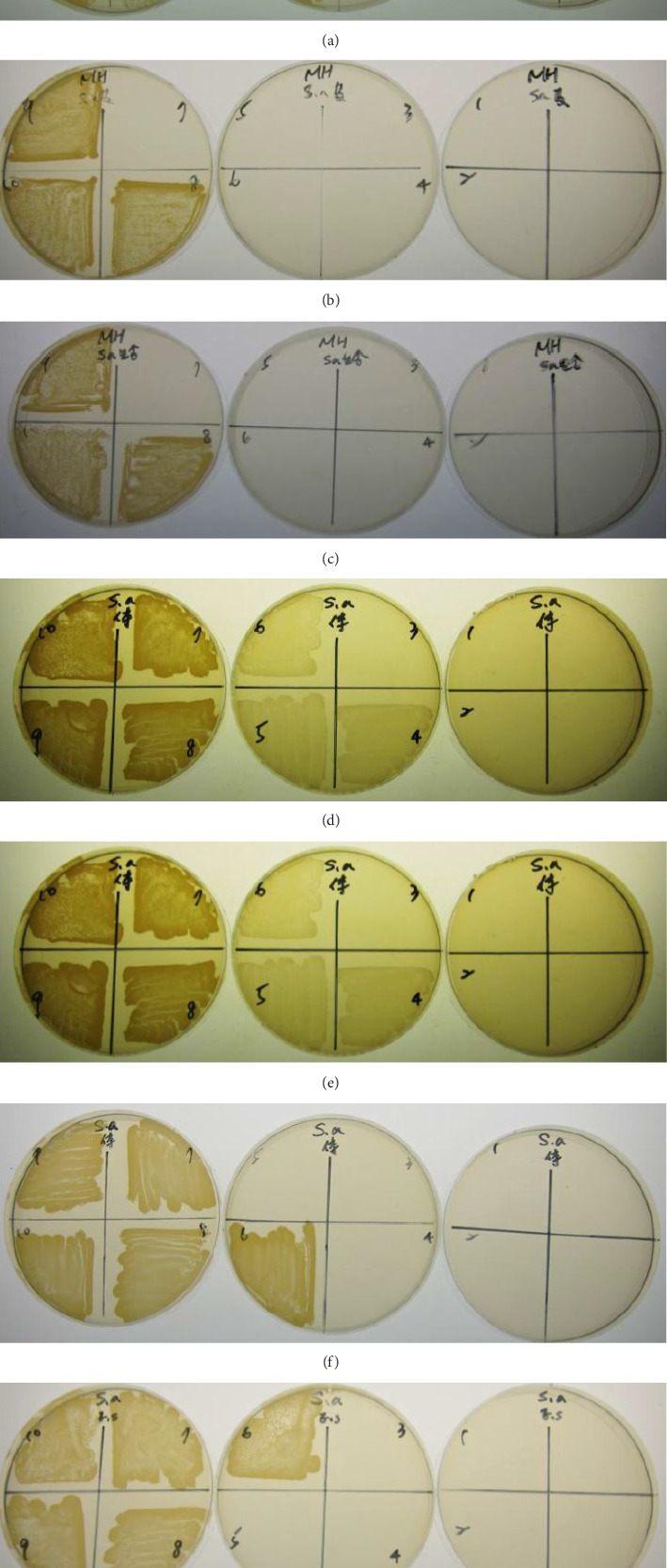
MBC testing of somatic and excretory-secretory antigenic proteins of adult and larval *Toxocara canis* on *Staphylococcus aureus.* (a) Intestine (TAI) and (b) body wall (TAC) of adult *T. canis*, genitals of (c) male (TMG) or (d) female (TFG) adult *T. canis*, (e) body fluid of adult *T. canis* (TAF), (f) larval somatic (TLS), and (g) excretory-secretary antigenic proteins (TES). Te: Tetracycline. Number labeled in the agar plate indicates: no. 1: 1:1; no. 2: 1:2; no.3: 1:4; no.4: 1:8; no. 5: 1:16; no. 6: 1: 32; no.7: 1:64; no. 8: 1:128; no. 9: 1:256; no. 10: 1: 512.

Regarding the MBC, the results of TAI, TAC, TMG, TFG, and TAF on *S. aureus* were 1: 16 (406.25 μg/mL), 1: 64 (101.56 μg/mL), 1: 64 (101.56 μg/mL), 1: 32 (203.13 μg/mL), and 1: 4 (406.25 μg/mL), respectively (Figures 4(a), 4(b), 4(c), 4(d), 4(e)) (Table 1). TLS and TES on *S. aureus* were 1: 16 (167.5 μg/mL) and 1: 16 (153.16 μg/mL), respectively (Figures 4(f), 4(g), Table 1).

### 3.5. Bactericidal Composition of Somatic and ES Antigenic Proteins of Adult and Larval *T. canis* on *S. aureus* in Contrast to Tetracycline as Assessed by HPLC

**Figure 5:**
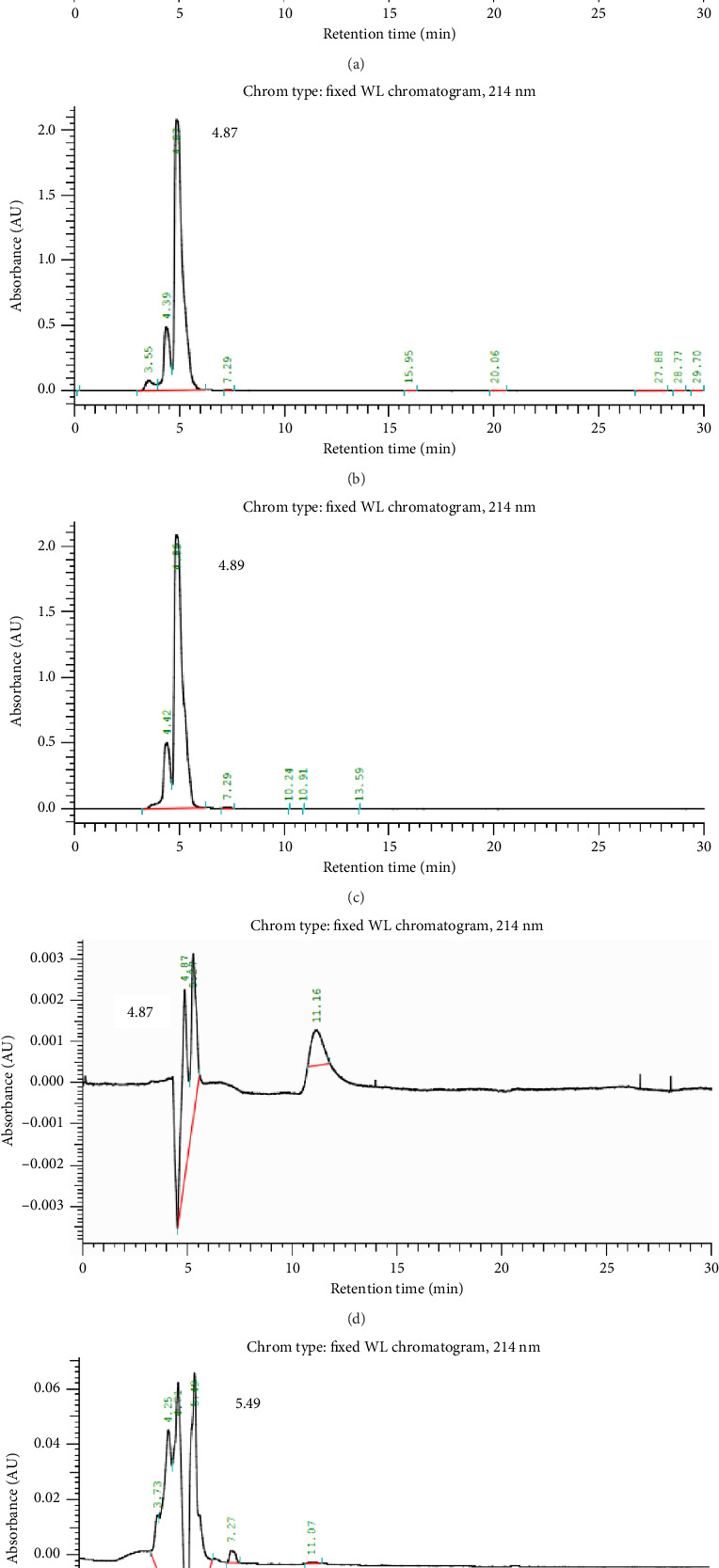
The bactericidal mechanism of somatic and excretory-secretory antigenic proteins of adult and larval *T. canis* on *S. aureus* in contrast to tetracycline as assessed by HPLC. (a) Standard antibiotic of tetracycline, (b) body wall of adult *Toxocara canis* (TAC), (c) genitals of male adult *Toxocara canis* (TMG), (d) somatic, and (e) excretory-secretory antigens of *Toxocara canis* larvae.

Standard tetracycline hydrochloride was prepared at a concentration of 500 μg/mL, and the concentration of various antigenic proteins was diluted with 100 X PBS to the following concentrations: TAC (130 μg/mL), TMG (130 μg/mL), TLS (32 μg/mL), and TES (30 μg/mL), and analyzed by HPLC. The results showed that control HPLC experiments of the tetracycline hydrochloride to test for residual antibiotics revealed that three peaks were detected, and the retention time of the major peak was 4.59 min (Figure 5(a)). The TAC had three peaks detected and, and the retention time of the major peak was 4.87 min (Figure 5(b)). TMG had two peaks, and the retention time of the major peak was 4.89 min (Figure 5(c)). Regarding the TLS and TES, three peaks and six peaks were detected, and the retention time of the major peak was 4.87 and 5.49 min, respectively (Figures 5(d), 5(e)).

### 3.6. MIC and MBC Assays of HPLC Eluate of Adult and Larval Antimicrobial Proteins With Molecular Weight Less Than 3 kDa

**Figure 6:**
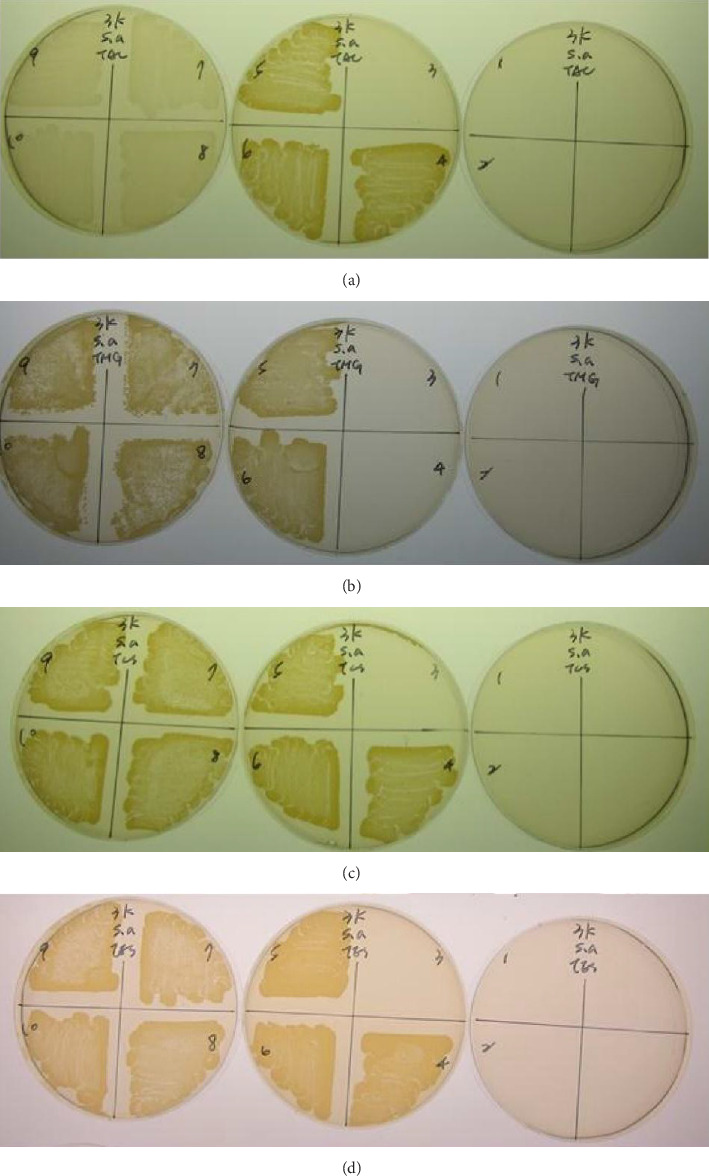
MBC assay of HPLC eluate of adult and larval antimicrobial proteins with molecular weight less than 3 kDa. (a) Body wall of adult *Toxocara canis* (TAC), (b) genitals of male adult *Toxocara canis* (TMG), (c) somatic, and (d) excretory-secretory antigens of *Toxocara canis* larvae.

The MIC results of TAC, TMG, TLS, and TES with molecular weight less than 3 kDa on *S. aureus* were 1: 8 (580 μg/mL), 1: 8 (673.75 μg/mL), 1: 4 (400 μg/mL), and 1: 8 (168.75 μg/mL), respectively (Table 1). Regarding the MBC, the results of TAC, TMG, TLS, and TES on *S. aureus* were 1: 4 (1160 μg/mL), 1: 8 (673.75 μg/mL), 1: 4 (400 μg/mL), 1: 4 (337.5 μg/mL), respectively (Figure 6, Table 1).

## 4. Discussion

This is the first report of antibacterial activity from *T*. *canis*, a parasitic nematode found in the small intestine of dogs. Several invertebrate species, including parasitic nematodes, have been found to produce compounds with antibacterial properties. *A. suum* and *Trichuris suis*, both intestinal parasites of pigs, have been shown to produce such compounds [14, 15, 21]. These elements constitute a primary humoral defense mechanism. Therefore, it is not unexpected that metazoan parasites residing in the gastrointestinal tract (GI) would produce antibacterial substances, given their presence in a microbe-rich environment with potential pathogens.

The preliminary findings of this study highlighted the antimicrobial potential of *T. canis* adult and larval somatic and ES antigens against *S. aureus*. Notably, the somatic antigenic proteins of adult *T. canis* worms, including TAC, TMG, TFG, and TAF, all exhibited effective antimicrobial potential against *S. aureus*. Similarly, larval somatic and ES antigenic proteins, TLS and TES, also demonstrated antimicrobial potential akin to the antigenic properties of adult worms against *S. aureus*. These findings are consistent with previous studies on *A. suum* and *T. suis* [13–15, 21], suggesting that parasitic nematodes may possess inherent antimicrobial properties that could be used for therapeutic purposes. Notably, different stages of *T. canis* worms including somatic adult, larvae, and larval ES antigenic proteins exhibited the same antimicrobial potential against *S. aureus*. This finding is very similar to a previous study by Midha et al. [23], which found that ESP from different *A. suum* life stages (in vitro-hatched L3, lung-stage L3, L4, and adult) and BF of adult males showed a bactericidal effect against *E. coli*.

This study also highlights the importance of understanding the bactericidal composition of these antigenic proteins. HPLC can be used to fractionate and analyze the components of a sample and is often used to identify specific substances within a mixture [24]. In predicting the bactericidal properties of unknown proteins, HPLC can provide insight by comparing the retention times of the unknown proteins with those of known antibiotics. If the retention times are similar, this may indicate that the unknown proteins have similar modes of action to the standard antibiotics [25]. In this study, we focused on HPLC-eluted proteins specifically from TAC, TMG, TLS, and TES because these antigenic fractions demonstrated the most consistent and potent bactericidal activity against *S. aureus* in preliminary susceptibility and MIC/MBC tests. Eluting only these components allowed for a more targeted HPLC analysis of their antimicrobial profiles and facilitated direct comparison with standard antibiotics such as tetracycline.

The present study showed that the retention times of the main peaks of TAC, TMG, TLS, and TES were like those of the antibiotic standard, tetracycline hydrochloride. This suggests that the bactericidal properties of these proteins may be like those of known antibiotics, which could have significant implications for the development of novel antimicrobial agents. Abner et al. [21] showed that the ES antigen (ESP) of adult *T*. *suis* was effective in killing gram-positive *S. aureus* and *E. faecalis*. Further HPLC analysis revealed that the composition of the ESP was similar to chloramphenicol, and it was speculated that its bactericidal mechanism may be like that of chloramphenicol.

Prokaryotes and eukaryotes naturally produce AMPs and are promising alternative antibiotics to combat multidrug-resistant microorganisms, Feurstein et al. showed that 62% of the 3828 AMPs studied consist of less than 40 amino acids and a molecular weight higher than 2.5 kDa. This suggests that the appropriate molecular weight of effective AMPs candidates is in the range between 2.5 and 4.4 kDa, since the average molecular weight of an amino acid is 110 Da [26], reflecting that the appropriate molecular weight of effective AMPs candidates is in the range between 2.5 and 4.4 kDa, since the average molecular weight of an amino acid is 110 Da [27]. The present study focused on HPLC-eluted proteins that were further processed to obtain a molecular weight less than 3 kDa, which was compatible with that of AMPs candidates with the range between 2.5 and ∼4.4 kDa. The proteins, whichever the author sees fit, showed the same bacteriostatic and bactericidal activity on *S. aureus* as the crude somatic or ES antigenic proteins of *T. canis* adult and larvae, indicating the existence of adequate AMPs in these low-molecular weight proteins that warrant further exploration.

The preliminary study provides valuable evidence of the antimicrobial properties of *T. canis* antigens, which could contribute to the development of new strategies to combat AMR. The results of the study are particularly relevant in the context of the global threat posed by AMR, as highlighted by the WHO and other health authorities [1–4]. By exploring the antimicrobial potential of parasitic nematodes [12], this research opens avenues for the discovery of new antimicrobial compounds that could be effective against drug-resistant pathogens. Further research is warranted to fully elucidate the mechanisms of action of these antigenic proteins and to assess their potential in clinical applications.

## Figures and Tables

**Table 1:** MIC and MBC of *Toxocara canis* adult and larval somatic antigenic proteins and larval ES antigenic proteins and antigenic proteins with a molecular weight less than 3 kDa eluted by HPLC on *Staphylococcus aureus*.

Symbol	MIC concentration (μg/mL)	MBC concentration (μg/mL)
TAI	1:32 (203.13)	1:16 (406.25)
TAC	1:64 (101.56)	1:64 (101.56)
TMG	1:64 (101.56)	1:64 (101.56)
TFG	1:64 (101.56)	1:32 (203.13)
TAF	1:8 (203.13)	1:4 (406.25)
TLS	1:64 (41.90)	1:16 (167.5)
TES	1:16 (167.50)	1:16 (167.50)

**Molecular weight less than 3 kDa antigenic proteins eluted by HPLC**		

TAC	1:8 (580)	1:4 (1160)
TMG	1:8 (673.75)	1:8 (673.75)
TLS	1:4 (400)	1:4 (400)
TES	1:8 (168.75)	1:4 (337.5)

*Note:* TAI: Intestine of adult *Toxocara canis*; TAC: Body wall of adult *Toxocara canis*; TMG: Genitals of male adult *Toxocara canis*; TFG: Genitals of female adult *Toxocara canis*; TAF: Body fluid of adult *Toxocara canis*; TLS: Somatic antigens of *Toxocara canis* larvae; TES: Excretory-secretory antigens of *Toxocara canis* larvae.

Abbreviations: HPLC, high-performance liquid chromatography; MBC, minimum bactericidal concentration; MIC, minimum inhibitory concentration.

## Data Availability

The raw data can be acquired upon request from the corresponding author.
